# Novel Optical Fiber-Based Structures for Plasmonics Sensors

**DOI:** 10.3390/bios12111016

**Published:** 2022-11-14

**Authors:** Zhi Wang, Wen Zhang, Xuecheng Liu, Muyang Li, Xianzheng Lang, Ragini Singh, Carlos Marques, Bingyuan Zhang, Santosh Kumar

**Affiliations:** 1 Shandong Key Laboratory of Optical Communication Science and Technology, School of Physics Science and Information Technology, Liaocheng University, Liaocheng 252059, China; 2 College of Agronomy, Liaocheng University, Liaocheng 252059, China; 3 Physics Department & I3N, University of Aveiro, 3810-193 Aveiro, Portugal

**Keywords:** optical fiber sensor, plasmonics, surface plasma resonance, special optical fiber structures, biosensors

## Abstract

Optical fiber sensors based on surface plasma technology have many unique advantages in specific applications such as extreme environmental monitoring, physical parameter determination, and biomedical indicators testing. In recent decades, various kinds of fiber probes with special structures were developed according to special processing such as tapering, splicing, etching, fiber balls, grating etc. In this paper, the fabrication technology, characteristics, development status and application scenarios of different special optical fiber structures are briefly reviewed, including common processing equipment. Furthermore, many special novel optical fiber structures reported in recent years are summarized, which have been used in various kinds of plasmonic sensing work. Then, the fiber-plasmonic sensors for practical applications are also introduced and examined in detail. The main aim of this review is to provide guidance and inspiration for researchers to design and fabricate special optical fiber structures, thus facilitating their further research.

## 1. Introduction

With the development of nanotechnology, nano-photonics has emerged as an important branch of photoelectric research. Here, the plasmonic sensor based on a kind of surface plasmons (SPs) has become one of most representative [1]. It is a kind of coherent oscillating element excited by photons or electrons on the surface of metal material. SPs were first proposed in the 1950s [2], and have two forms according to different conditions: propagating surface plasmon polaritons (SPPs), as well as localized surface plasmon polaritons (LSPs) [3]. SPPs are surface electron oscillations that propagate along with the thin metal layer on metal surface. When the SPPs interact with evanescent waves (EWs) produced by p-polarized light, the total reflection propagation phenomenon is produced. Here, the surface plasmon waves (SPWs), which are essentially electron density waves, will excite. The direction of SPWs follows the metal surface, on which a strong electromagnetic field will be produced if the wave vector of the incoming light matches with the wave vector of the SPWs. Under this condition, the intensity of reflected light decreases significantly. This phenomenon is what we call surface plasmon resonance (SPR) [4]. When the size of a nanomaterials wavelength is significantly smaller than that of EWs on the surface, the electronic oscillation will be restricted to the surface of nanostructures. And the localized surface plasmon resonance (LSPR), with a highly localized electromagnetic field amplification phenomenon at a particular resonance wavelength, will occur at the outside edge [5] of materials with a few nanometers-structure [6]. The plasmonic sensor based on SPWs is sensitive to the distribution of the refractive index (RI) on both sides of the interface. Here, the depth of the field penetration determines the intensity of the localized characteristics of the field, as well as the sensing range of the surrounding media that plays a decisive role in the sensitivity of the sensor. The first romantic meeting between SPR and sensing technology was suggested in 1983 by Liedberg et al., who designed sensors for the real-time detection of gas and quantitative monitoring of selective anti-body reactions. What is more, they boldly predicted the bright future of the plasma sensor [7]. As a new research field, research on the SPR sensor is attracting more and more attention and has been exploded as a new subject. One of the earliest examples is the prism-based SPR sensor. However, this special kind of sensor system seems usually enormous and has numerous blemishes, including high production costs, limited anti-interference capability, complicated optical elements, inconvenient handling, and difficult remote measurement [8].

The SPR effect, which was firstly proposed by Jorgenson et al. in 1993, was demonstrated based on coating the bare optical fibers with a gold film [9]. Subsequently, the sensor based on the optical fiber waveguide gradually replaced the prism structure due to its benefits of small size, simple structure, simplified packaging, low cost, strong portability, remote detection and on-site monitoring [10]. The propagation of light is mainly in the core of the fiber, following the rule of total internal reflection (TIR) [11]. By changing the properties and materials of optical fiber, many different types of fiber (glass and different polymers) [5] can be designed and used to realize the modulation of the transmitted optical wave power, wavelength, phase and frequency. For this, the perception of external subtle changes is then realized through data demodulation and processing. According to these steps, it can be applied to detect different biomolecules [12], chemicals [13], and temperature [14], and also a wide range of other applications such as the water environment pH value and RI [15], various gases [16], humidity [17] etc. However, with the increasing demand for the application of sensor technology, especially in life science, clinical diagnosis, medicine, and food safety, the sensitivity and specificity of plasma sensors for low-dose analyte detection are required to be higher [18]. Therefore, improving the sensitivity and detection range has been the focus of SPR/LSPR technology in the detection system. The SPR/LSPR-based fiber analyte system commonly consists of a light source, fiber probe, and spectrometer. SPs are excited at the metal layer and the sensing layer interface by the EWs produced by the light flux. The light wavelength, probe geometry, fiber parameters, and characteristics of the metal layer all play a significant role in the maximum coupling efficiency between the evanescent field and SPWs. In light of the aforementioned issues, the two most efficient strategies to enhance the sensing performance of SPR/LSPR sensors are to optimize both the structure of the fiber waveguide and immobilization effects of the coating materials [19].

Based on the point of view of optimizing the structure of the fiber waveguide, two basic approaches can be considered. The first one is using a special type of optical fiber as a probe to change the characteristics of the inner guiding wave. And the second one is employing special treatment and processing toward the structure of the optical fiber to change the guiding wave geometry [18]. Since the widespread usage of optical fiber, multi-mode fiber (MMF) and single-mode fiber (SMF) as a common fiber structure were employed to effectively reduce the cost of sensing systems. In contrast, through special fabrication processes or doping materials, many special fiber structures with fabricating characteristics, a controllable mode, and easy integration [20] (such as polymer optical fiber (POF) [21,22], multi-core fiber (MCF) [23], photonic crystal fiber (PCF) [24,25], and hollow core fiber (HCF) [26,27], among others) have been designed and fabricated for a variety of plasmon detection applications. Since the incident light propagates in the core of the fiber, which is completely confined inside the cladding, it is difficult for EWs to reach the cladding surface. For this reason, it is usually essential to change the geometry of the optical fiber structure by a special processing method to break the original optical transmission mode. Later, more and more fiber structures, such as tapered optical fiber (TOF) [28], D-shaped [29], U-shaped [30], and the fiber grating structure [29,31], have appeared on the stage. These different optical fiber structures need to be realized by special corresponding optical fiber processing equipment.

**Figure 1:**
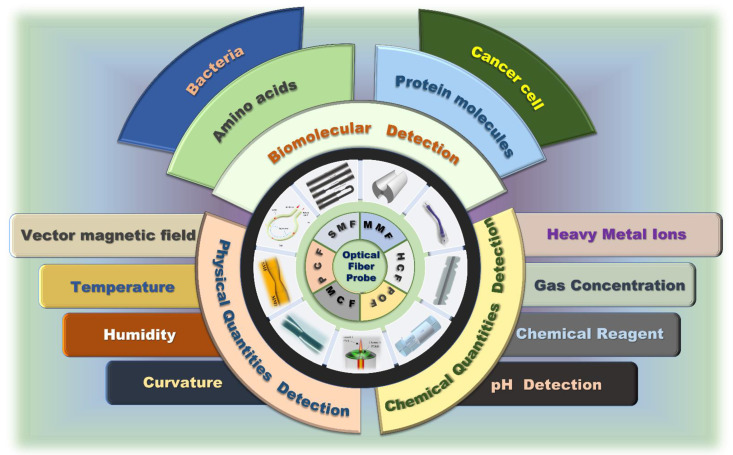
The special optical fiber structure is widely used in the plasma sensing system [32,33,34,35,36,37,38,39,40].

As we can concluded in Figure 1, this study mainly focusses on the introduction and summary of the SPR/LSPR technology-based sensor structure of recent years. Based on the fabrication technology and sensing principle of the classical optical fiber platform, the multi-parameter detection and sensing performance improvement of the novel special optical fiber structure are reported in detail. Then, practical applications based on fiber-plasmonic sensors are also introduced and analyzed at this point. Finally, future evolution directions in the fiber optic sensing system based on plasmonic technology are identified, which are convenient for researchers to conduct research and development work. 

## 2. Processing Technology

In this section, several kinds of common fiber-optic processing are introduced and shown in Section 2.1. Based on these, many classical special optical fiber structures will be discussed in detail in the following summaries. Many researchers have developed the above-mentioned optical fiber structures discussed in this section and summarized in this manuscript to make it easier for readers to understand the related research progress.

### 2.1. Optical Fiber Processing Technology

Laser processing as the most popular application mainly involves four laser writing technologies according to different heating lasers: ultraviolet laser (UV laser) [41,42], excimer laser [43], femtosecond laser [31] and CO_2_ laser [44], that can fabricate the specific fiber grating structures, grooving, and heating in optical fibers with different characteristics based on specific needs. At present, the most popular treatment methods widely used in fiber grating coupled SPR sensors are CO_2_ lasers. The fabrication of fiber gratings by CO_2_ laser irradiation is mainly caused by the residual stress release or physical deformation caused by the CO_2_ laser pulse periodically heating the fiber, which results in the RI modulation of the fiber. Further, due to the limitation of CO_2_ laser focusing ability, it can also replace the electrode heating technology to fabricate the TOF structure.

Arc discharge technology is a usual method to melt the optical fiber layer materials by periodically heating the controlled electrode, freely adjusting the current and precisely restricting the discharge time. This method is not suitable for fabricating FBG structures because of its large arc discharge area and electrode power loss caused by continuous discharge. However, it is widely used in fabrication processes such as spherical structures [45], tapered structures [45,46], fiber fusion splicing [47] etc.

The arc-shaped groove polishing method is usually used in large-length polishing and batch polishing. The wheel-type side polishing and grinding method has the advantages of simple operation, good repeatability, large length optical fiber polishing, low processing cost etc., and has been widely used in laboratory research. Additionally, the expansibility of the equipment is strong, and the fixed axis system can be added to realize the fixed axis polishing function. Thus, a more complex plane structure that is not easy to break can be made on the premise that the structure of the optical fiber remains flat. Because the diameter of the optical fiber is very small, it needs to be placed carefully and reasonable parameters set; otherwise, it may induce fiber fracture in the processing, or polishing of surface roughness, and other quality problems.

Oxyhydrogen flame spraying technology is a classical fabrication process of tapered fiber structure, which is simple to operate and low in cost, and can be controlled by computer program. The entire production process is described in subsequent sections. While the exposed fiber is heated, the two sides of the tractor are controlled by the motor and computer to maintain a constant stretching speed and rotation speed, so as to ensure that the heated area can be uniformly stretched at a constant temperature. But this method requires a long preheating process to achieve the softening temperature of the fiber, and it is greatly affected by external environmental factors so that it is difficult to keep the heating temperature constant.

**Figure 2:**
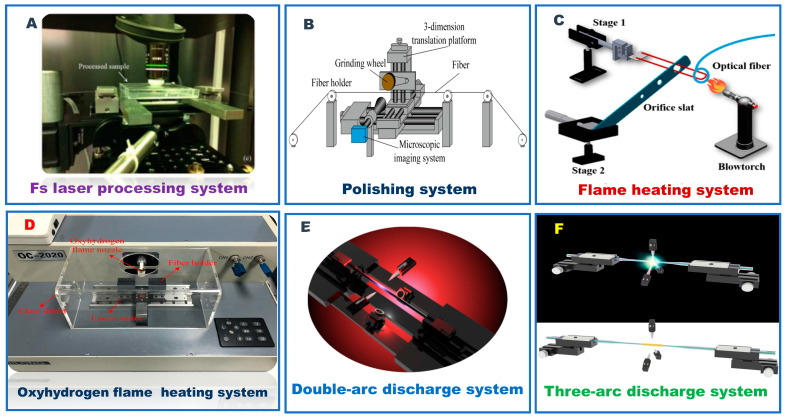
Schematic of several kinds of common fiber-optic processing. (**A**). Fs laser processing system. Reprinted with permission from *Optics and Lasers in Engineering*, Copyright 2019, Elsevier [26]; (**B**). Polishing system. Reprinted with permission from *Sensors*, Copyright 2021, MDPI [48]; (**C**). Flame heating system. Reprinted with permission from *Polymers*, Copyright 2021, MDPI [49]; (**D**). Oxyhydrogen flame heating system. Reprinted with permission from *Optics & Laser Technology*, Copyright 2020, Elsevier [38]; (**E**). Two-arc discharge system. Refer to [50] (**F**). Three-arc discharge system. Reprinted with permission from *Optics Express*, Copyright 2021, Optica [51].

In the chemical etching method of optical fiber processing, hydrofluoric acid solution is usually employed for etching the cladding surface or even the part of the fiber-optic core, and the etching depth of the cladding is adjusted by controlling the etching time. Because this method is applicable to any type of optical fiber without any expensive instruments, it has the superiority of low production cost and operation difficulty. However, during the corrosion process, the concentration of hydrofluoric acid will gradually decrease, which will affect the corrosion rate. Thus, it is a big challenge to accurately control the corrosion depth. At the same time, the corrosion process only occurs on the cladding, as well as the coupling between the cladding mode; a core-based mode is insufficient. Finally, the corroded fiber surface may have residue, which makes it difficult to ensure the mechanical strength. It needs to be thoroughly and carefully cleaned, so as not to affect the subsequent experiments. Figure 2 is illustrating several common fiber-optic processing systems.

### 2.2. Fiber Grating

The fiber grating is produced by irradiating the fiber with a certain wavelength and intensity of laser and exciting the RI of the fiber core. It uses the photosensitive optical fiber and is an important part of the optical fiber. It has excellent performance and wide SPR applications. According to the different grating periods, fiber gratings can be divided into long-period fiber gratings [52] and short period fiber gratings [53]. When the optical signal passes through the fiber grating, the optical signal of a specific wavelength will be coupled into the cladding. When the optical signal passes through the fiber grating, the optical signal of a specific wavelength will be coupled into the cladding, so that the in-plane wave vector of the incident optical wave at the dielectric metal interface increases, until it meets the resonance condition matching the propagation constant ($K_{SP}$) of the surface plasmon polariton. At this time, the surface plasmon resonance signal is generated at the interface of the metal film. The matching condition expression is as follows:

(1) $$\begin{array}{c} K_{SP}=K_{inc,x}+mK \end{array}$$

$K_{inc,x}$, here, is the diffraction order of the incident light wave projected along the *X*-axis, and also the integral multiple of the grating wave number *K*.

Long et al. [54] systematically analyzed the sensitivity limit of the grating coupled configuration plasmonic sensing system from a theoretical point of view. It is reported that the sensitivity limit is close to the detection wavelength and the grating period. The regular can be obtained by increasing the corresponding parameters, the sensitivity limit of the sensor is increased. Additionally, the grating depth and duty cycle are also important directions of optimization [55].

In order to improve the SPR sensing performance, the traditional processing method of the fiber grating structure is usually fine-draw tapering [56] and etching [57]. However, this method can very easily damage the stability of the fiber structure, which affects the coupling of the cladding modes. For solving the above problems, the tilted fiber Bragg grating (TFBG) is used as the sensing structure to stimulate the SPR effect, and the tilted periodic disturbance in the fiber core is used to promote some light to diffract into the cladding [53,58,59].

### 2.3. Taper Optical Fiber

The TOF structure can be obtained by heating the fiber to the molten state at high temperature with physical stretching. The taper structure is a good choice to ensure the field interactions between the EWs and SPWs. Fiber taper variation refers to the change in the shape of the original fiber after taper variation treatment, which changes its optical characteristics. Because of its compact structure and simple fabrication, adiabatic fiber taper has become a research hotspot, which is mainly used to prepare various optical sensors. The guided wave leaking from the core attenuates exponentially from the tapered waist area to the outer surface of the cladding section. This structure is widely used in the optical fiber coupler, optical fiber sensor, and other fields. At present, there are three main methods to prepare the tapered fiber probe: physical polishing, chemical etching, and fused tapering. The fused tapering method is a method to produce localized deformation by heating the optical fiber. The heating sources include traditional oxyhydrogen flame heating, arc discharge, CO_2_ laser, resistance heat source, etc. As the heat source, the CO_2_ laser can achieve extremely high control accuracy, and the radial size after laser focusing is only a few hundred microns. However, the cost of using high-power lasers to manufacture fiber structures is relatively high. At the same time, it is hard to ensure the uniform radial heating of the optical fiber heating part.

### 2.4. Fiber Ball

The micro-ball optical fiber structure is made by heating the end face of the spinning optical fiber into melt statues, and then condensing it into a spherical structure through air under the action of centrifugal force. Due to the heating process, it is necessary to provide more stable heating power and a more uniform heating environment; the electrode heating method is often used to prepare this special structure. Taking the SMF structure as the example [18,19,20], the principle is shown as follows. The RI inside the microsphere is higher than the surrounding environment, so the light inside the spherical resonator follows the principle of TIR and is constantly reflecting back into the sphere. When some of the light reaches the curved edge and leaks out slowly from inside the sphere, this will make the EWs propagate along the surface and decay exponentially to escape into the surrounding environment. Here, the structure core radius of the evanescent field is closely related to the incident light wavelength. When the analyte interacts with the stationary object on the spherical surface, the effective RI of the light field around the probe will change. The interaction between the sample and light is enhanced by the confinement and rebound of the light in the cavity, which eventually causes the exposure to the environment change to lead to the change in spectral properties, such as the shift in wavelength and frequency. The fiber-ball structure has such superiorities like simple fabrication, high mechanical stability, and strong reproducibility. Further, it is very sensitive to change in surface tension or stress, which can be the reason that this structure is very suitable for the detection of physical parameters.

### 2.5. Hetero-Core Fiber Structure

As a common and popular method to fabricate optical fiber sensors, the fusion method can break the binding of the inherent transmission mode of TIR of the fiber core and cladding. Thus, the fiber probe can be fabricated with high sensitivity, small detection limit, strong anti-interference ability etc., to use in different complex environments or even extreme environments [60]. Common types of fiber splicer machines are the FSM100+, FSM-60S, and Furukawa S178A. These splicers are not only able to perform normal splicing, but also can be used for fusion, and so on; in addition, over-splicing [61] and dislocation splicing [61] of optical fiber structure can be performed. The fusion mode results in a core mismatch between different types of optical fibers, which can result in partial light leakage and excites different high-order cladding modes near the fusion point. Interference is caused by the mismatch of the effective optical path difference and fiber diameter between different modes. These modes will eventually be spliced into the next portion of the fiber [62]. In theory, the multistage fusion structure is actually an inline Mach–Zehnder interferometer (MZI) [63]. It is the mode interference between different optical fibers that produces the interference spectrum. When the object and parameter of the environment change in different degrees, the RI of the surrounding environment will change in different degrees, which results in a change in the peak wavelength of the interference spectrum, laying a solid foundation for optical fiber sensor research. The core offset structure, as part of the sensing unit, functions as a mode converter for energy exchange between the cladding mode and core mode, while the cascaded sensor has the capability of simultaneously measuring many parameters [64]. In fact, so far, researchers have developed optical fiber sensors based on different fusion modes and achieved good sensing performance.

### 2.6. D-Shape Fiber

The general D-shaped optical fiber is made directly by the manufacturers [65]. However, if only the cladding of the optical fiber is polished, the internal core structure does not change, which is called the side polishing optical fiber structure.

This is the most direct way to place the core mode as close as possible to the special metal mode and external media mode, so as to more effectively stimulate the SPWs. That is because when the cladding thickness of the fiber is small enough, usually only a few microns away from the core, by polishing or chemical etching, the “leakage window” of the field of EWs will be easily satisfied. Thus, it is applied to the optical fiber sensing system, which excites the SPWs. Side-polished fiber devices can control the region of evanescent field and adjust the unique optical characteristics and reduce the package size, which allows D-shaped fiber to be widely used. It should be noted that the length of the polished area, minimum distance from the fiber core to the polished surface, and roughness of the polished surface and coating material are important factors affecting the transmission characteristics of polished fibers.

### 2.7. S-Shape Fiber

The S-shaped fiber structure, first proposed by Rui yang et al. [66] for use as a miniature MZI, is unique in that it bends at the taper transition of the excited high-order mode. The transmission characteristics of the fiber structure are affected by the structure parameters such as axial offset length, waist diameter, bending degree, and so on. The above parameters are mainly related to the tensile length and axial offset of the fiber. Therefore, thorough, in-depth research can be conducted on the influence of different stretching lengths and axial offset lengths on the transmission characteristics of the sensor.

Other interesting phenomenon is the extinction ratio decreases with the increase in the core offset. The reason for this change is that a certain axial deviation will break the symmetry of the structure and promote the excitation of higher-order modes. When the axial offset is too wide, however, the majority of the energy is wasted as bending loss, and the energy of the basic mode and higher order mode participating in the interference process declines. The transmission spectrum will be affected by the structure’s transverse stretching. It indicates that a lateral offset that is too high or too little will result in insufficient excitation energy.

### 2.8. U-Shaped Fiber

The fabrication process of the U-shaped fiber-optic probe is simply described as fixing the cladding layer of optical fiber with a specific fixture to the designed shape and then heating it with a flame, and then the ring will shrink into a “U”-like shape and be permanently fixed [49]. U-shaped optical fiber has high sensitivity and point illumination, and its bending structure can reduce the difficulty of fixed materials.

In the theoretical analysis of the U-shaped probe structure, the bending of the fiber is usually considered to lead to the gradual change of the incident angle of the light, which improves the sensitivity of the sensor. This is because the smaller the incidence angle, the greater the depth of penetration of light; thus it can increase the intensity of the evanescent field [67]. The intensity of the evanescent field of this structure is restricted by the critical angle of incident light, which does not increase with the decrease in bending radius. When the structure is too bent, the sensitivity of the sensor drops sharply. On the other hand, when the bending radius is determined, the sensitivity of the sensor increases with the increase in the sensing length. Therefore, special tests are usually required to determine the optimal bending radius and bending length [68]. The low-order SPP mode excited by the U-shaped fiber structure is on the side far from the bending center, and the coupling between the low-order SPP mode and guide mode of the fiber is easier and stronger than the high-order SPP mode. The loss caused by the coupling is the main part of the SPR loss peak, so only one side of the fiber can be coated (first bent into a U-shaped fixed film and then coated), so that the fiber need not be rotated, thus avoiding a fiber break, reducing the difficulty of the process.

**Figure 3:**
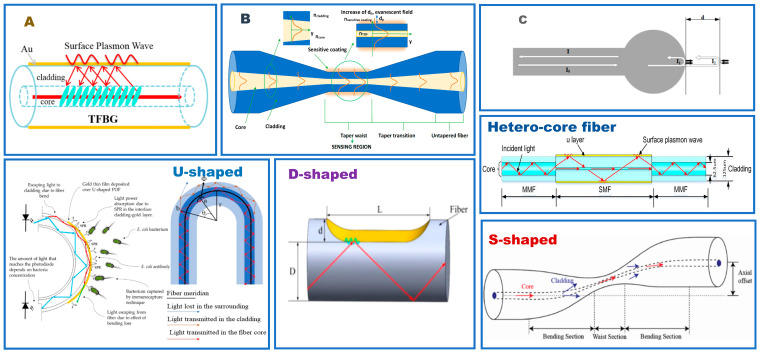
Schematic diagram of several classical special fiber structures. (**A**). TFBG. Reprinted with permission from *Sensors*, Copyright 2017, MDPI [59]; (**B**). TOF. Reprinted with permission from *Sensors*, Copyright 2022, MDPI [70]; (**C**). Micro-ball Reprinted with permission from *IEEE Trans Nanobioscience*, Copyright 2020, IEEE [71]; (**D**). Hetero-core fiber. Reprinted with permission from *Biosensors and Bioelectronics*, Copyright 2019, Elsevier [72]; (**E**). U-shaped. (**a**) Reprinted with permission from *Sensors*, Copyright 2020, MDPI [73]. (**b**) Reprinted with permission from *Optical Fiber Technology*, Copyright 2020, Elsevier; [35]; (**F**). D-shaped. Reprinted with permission from *Sensors*, Copyright 2021, MDPI [48]; (**G**). S-shaped. Reprinted with permission from *IEEE Access*, Copyright 2021, IEEE [74].

The U-shaped optical fiber sensor uses the following two sensing modes to realize the above sensing principle. If only the coating layer is removed and the fiber is bent into a U-shape by heating or fixing on the mold, then the TIR condition of the fiber core is no longer satisfied, as shown in Figure 3(Ea). The light will come out from the fiber core into the cladding layer, where part of the light is refracted to the outside, and part of the light is reflected at the cladding–external environment interface, generating EWs that can affect the metal film plated on the fiber. The cladding of the fiber can also be removed before bending, and a kind of U-shaped fiber sensor with a simpler principle will be obtained. It can be concluded that, the evanescent field generated by TIR in the fiber core will be directly used to stimulate SPW. Then, the U-shaped structure changes the original fiber core transmission mode, and the effective evanescent absorption coefficient increases with the decrease in the radius of the curvature [69].

Figure 3 shows the schematic diagram of several classical special fiber structures. The wide application of special fiber structures in the last four years are collected in Table 1.

## 3. Novel Plasmonic Fiber Structure

### 3.1. Polymer Optical Fiber-Based Novel Structure

Optical fiber made of light transmitting polymer can be used as an SPR sensing probe. The major purpose is to shift the resonance wavelength of SPR to a longer wavelength. In general, the analyte’s RI is strongly connected to the fiber core’s RI, and the resonance wavelength at high sensitivity is always larger than that at low sensitivity. The sensitivity is relatively weak in the lower RI detection range.

As the surrounding RI gradually approaches the core RI, the speed at which the sensitivity can be accelerated increases, thus adjusting the resonant wavelength of SPR to a longer wavelength. The diversity and ease of doping of polymer fiber materials characterize optical fiber composed of light transmission polymer. As a kind of fine RI adjustable fiber, it can greatly improve the sensing performance. Additionally, POF is very suitable for detecting biomolecules and industrial environments as an SPR/LSPR sensor due to its simple fabrication, excellent mechanical properties, strong anti-interference ability, and good biocompatibility.

Liu et al. [98] presented a POF-based SPR sensor for simultaneous measurement of RI and temperature. The double-U-shaped probe is made by polishing both sides of the POF. A coating of gold film is deposited on both sides of the polished surface, and a layer of polydimethylsiloxane is coated on one side. The polished POF is prepared using a grinding wheel polishing system. The POF is fixed on the optical fiber bracket. The POF is grinded with a rotatable grinding wheel covered with abrasive paper. The speed is controlled by the computer. The double-sided polished probe has an enhanced SPR effect in the external medium, providing a larger area for the measured sample. The parallel polishing structure is of great benefit to the development of multi-parameter measurement sensors. The wavelength shift of two resonance peaks may be used to concurrently estimate RI and temperature. The experimental findings reveal that the sensor’s RI sensitivity is 1174 nm/RIU, RI range is 1.335–1.37, and temperature sensitivity −0.7 nm/°C in the 30–80 °C temperature range.

**Figure 4:**
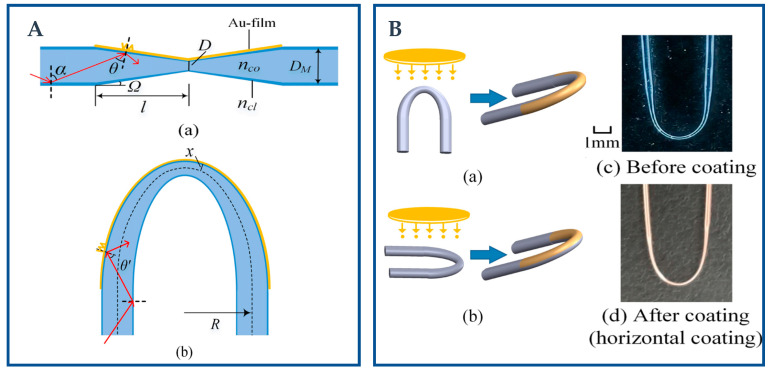
(**A**). Schematic diagram of the (**a**) straight and (**b**) U-shape of TPOF. Reprinted with permission from *Optik*, Copyright 2022, Elsevier [19]; (**B**). The schematic diagram of the coating results in the U-shaped probe based on POF (**a**–**d**). Reprinted with permission from *Optik*, Copyright 2022, Elsevier [19].

Teng et al. [99] presented a POF-based SPR sensor for measuring the RI and liquid level at the same time. The probe is made by drilling equidistant holes along the optical fiber axis on the polished POF, and a layer of gold film is deposited in the polished area to obtain the SPR probe. The wavelength location and depth of the SPR peak can be used to identify changes in the RI level. The range of the RI was 1.335–1.40, sensitivity 2024.41 nm/RIU, probe resolution 5 mm, and level measurement range 25 mm. Teng et al. [19] proposed an SPR sensor based on a U-shaped tapered POF (TPOFs). The schematic diagram of a straight and U-shaped TPOF is shown in Figure 4A. By controlling the displacement and heating time, TPOF with different taper ratios can be prepared. A layer of gold film can be deposited at the top and side of the U-shaped probe bend using the plasma sputtering device. The coating results are shown in Figure 4B. The sensitivity of the U-shaped probe is 1534.53 nm/RIU when the taper ratio is 6.7 and the RI sensing range is 1.335–1.41. It has potential application prospects in the field of biochemical sensing.

### 3.2. Multi-Core Fiber-Based Novel Structure

MCF has been used in the communication field as a mature technology because of its advantages of low loss, space division multiplexing and excellent mechanical properties [100]. Because these unique advantages, MCF are also very suitable for optical fiber sensors; therefore, MCF has become increasingly popular in the field of optical fiber sensing in recent years. In fact, when light propagates in the MCF, it has less transmission loss than SMF and MMF, and is extremely sensitive to small RI changes in the environment around it [101,102], which makes MCF more competitive in the field of optical fiber sensing. In normal MCF, due to the different RI of the cladding and core, the light transmits in the fiber through TIR; thus, it is necessary to obtain EWs by means of tapered, convex, and eccentric core structures. The evanescent field, together with the noble metal films or nanoparticles, lays the foundation for SPR and LSPR excitation.

**Figure 5:**
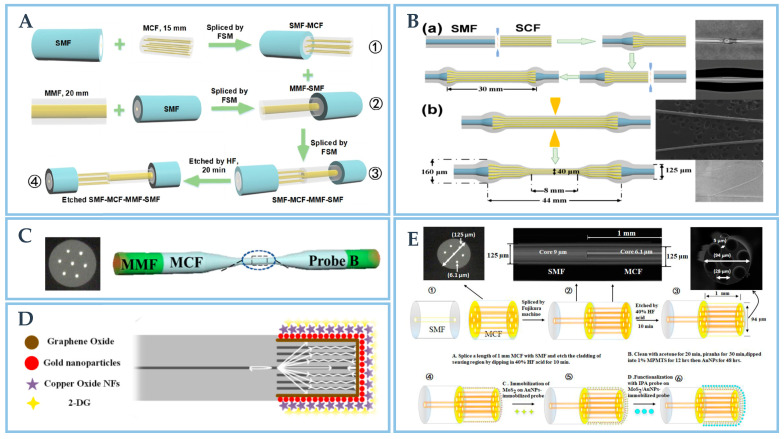
(**A**). The SMF-MCF-MMF-SMF structure fabrication process. Reprinted with permission from *Optics Express*, Copyright 2021, Optica [50]; (**B**). Fabrication steps of (**a**) CSC based sensor structure, (**b**) CTC based novel sensor structure with the scanning results using CCD camera. Reprinted with permission from *Optics Express*, Copyright 2022, Optica [103]; (**C**). MMF-MCF structure. Reprinted with permission from *IEEE Transactions on Instrumentation and Measurement*, Copyright 2022, IEEE [104]; (**D**). The sensing schematic of MCF-SMF structure. Reprinted with permission from *Biosensors and Bioelectronics*, Copyright 2020, Elsevier [105]; (**E**). The CCD scanning results of etched-MCF-SMF with the fabrication process. Reprinted with permission from *Journal of Lightwave Technology*, Copyright 2021, IEEE [101].

In order to detect the concentration of creatinine in human body, Li et al. [50] designed a SPR fiber optic sensor based on SMF-MCF-MMF-SMF structure. The sensor uses SMF, MMF and MCF to develop online MZI for new optical fiber sensor structures. The fabrication steps of the optic-fiber sensor structure are shown in Figure 5A. The sensor is fabricated by etching the hetero-core structure fiber-optic structure with hydrofluoric acid. The diameter of the probe after etching is 90 μm, compared with the probe diameter before etching, the evanescent field on the probe surface is greatly enhanced. The sensor can specifically detect creatinine in the linear range with the help of a variety of two-dimensional materials and creatinine enzyme.

Based on the work, Li et al. proposed and designed a novel LSPR sensor to detect creatinine in aquaculture [103]. The related schematic diagrams of probe structure are shown in Figure 5B in detail. This sensor uses heterogenous core mismatch and tapered probe structure (i.e., convex fiber tapered seven-core fiber convex fiber (CTC) structure) to increase EWs. This sensor combines fusion and tapered structure, which provides a reference method for novel fiber structure. Similarly, Zhu et al. [106] designed an fiber-optic temperature sensor with high sensitivity based on SPR. The structure is made of single-mode two-core fiber (TCF) polished into a circular truncated taper. Because of the high RI sensitivity of SPR sensor and the high thermal light coefficient of polydimethylsiloxane layer (PDMS), the sensor can realize figure of merit (FOM) up to 0.034/°C. The temperature sensor has potential applications in the fields of biomedicine and biomaterials. Zhu [104] et al. designed a sensor structure based on MMF and MCF for acetylcholine detection, the structure is shown in Figure 5C. As shown in Figure 5D, Singh [105] et al. designed a small portable sensor based on LSPR to detect different types of cancer cells. The sensor probe is spliced by MCF fiber and SMF. Additionally, Kumar [101] et al. designed an optical fiber sensor based on LSPR to detect *Shigella*. The sensor probe is made by splicing MCF and SMF and then etching as shown in Figure 5E.

### 3.3. Hollow Core Fiber-Based Novel Structure

**Figure 6:**
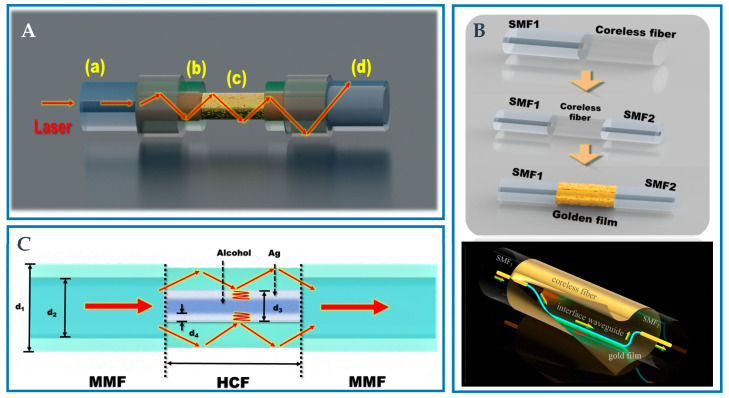
(**A**). The schematic of the sensor probe and each section of the probe: (**a**) single-mode fiber, (**b**) capillary fiber, (**c**) capillary fiber filled with ultraviolet curable adhesive, (**d**) step-index multimode fiber. Refer to [109]; (**B**). The structure fabrication process and schematic diagram of the etched structure. Reprinted with permission from *Optics Letters*, Copyright 2019, Optica [110]; (**C**). Schematic diagram of the MMF-HCF-MMF structure. Refer to [111].

The construction of HCF includes a unique central pore and an external cladding. As a kind of novel optical waveguide, HCF not only has a simple structure and low loss, but also has good flexibility and security. Therefore, HCF has been widely used in many fields as an ideal substitute fiber. The SPR/LSPR sensor based on HCF adopts wavelength detection, which is very sensitive to the change in the RI of the external environment. The incident light is produced by a broad-spectrum light source, and the relationship with the RI of the object is obtained by observing the position of the resonant wavelength in the transmission spectrum. Additionally, HCF has a large numerical aperture, which allows higher-order modes to make more contributions to sensitivity and further improve the performance of the sensor. In the research, people often fuse HCF and ordinary optical fiber together, and fill HCF with a solution or metal material to realize SPR RI sensing. Because of its simple structure, low cost, and minimal loss in visible and infrared areas, HCF has been actively explored as a suitable replacement fiber [107]. When the light propagates inside the hollow core, it will form EWs on the inner surface of the metal film, which will stimulate free electron oscillation, thus generating SPWs. SPWs propagation constants are greatly dependent on the RI of the surrounding medium. Therefore, even if the change in the RI of the measured material is very small, the wavelength of the incident light wave when coupled with SPWs will still change. Therefore, the most effective way to measure the RI of a material is to measure the coupling wavelength of the light wave and SPWs [108]. Additionally, HCF has a large numerical aperture, which allows the high-order mode to further improve the performance of the sensor. Liu et al. [109] presented a core RI control-based RI sensor. A unique fiber with changeable core RI was created using UV curable adhesives with varying RI and HCF. Using this special optical fiber, a novel RI SPR sensor with a controllable RI detection range is realized. The schematic of the sensor probe is shown in Figure 6A.

This research presents a novel method for detecting high RI solutions as well as a novel strategy for the manufacturing of SPR sensors. Zhang et al. [110] proposed and realized a new SPR structure by combining femtosecond laser writing waveguide technology with the SPR effect, which is used to measure the RI of analytes, as shown in Figure 6B. In the coreless fiber, a special U-shaped wave near the fiber surface is etched, which can generate a strong EWs field sensitive to the surrounding medium and trigger the SPR effect on the fiber surface. The optical fiber surface is coated with a 45 nm thick gold sheet to create a perfect optical fiber interface waveguide SPR sensor. Zhou et al. [111] proposed an SPR temperature sensor based on HCF filled with alcohol, which has good biocompatibility and stable performance. The design structure is shown in Figure 6C. The sensor has good temperature sensing capabilities because of the high RI sensitivity of the SPR effect and high thermal optical coefficient of alcohol. The linear sensitivity is up to 1.16 nm/°C in the range of 35.5~70.1 °C. The sensor requires a small volume and high sensitivity, and is suitable for temperature detection in biological and chemical reaction processes. Zhang et al. [112] developed a dual channel SPR sensor based on HCF for simultaneously measuring liquid RI and ambient temperature. The two channels are coated with different materials to realize the measurement of liquid RI and ambient temperature. The fabrication method is well discussed in the work and the proposed sensor has high sensitivity in a wide measuring range.

### 3.4. Photonic Crystal Fiber-Based Novel Structure

PCF is a special optical fiber, which has holes arranged in a specific way, and uses a specific channel structure with a constant longitudinal RI to guide the transmission of light. Generally, the optical properties of PCF can be changed by changing the size and shape of the PCF holes. Here, the SPR-based PCF is coated with gold film in the specific air holes. The EW in PCF can be controlled by a specific hole array, and the SPR effect is excited when a specific electromagnetic wave interacts with the free electrons on the metal surface. At the same time, by filling characteristic materials, the application scenarios of SPR-based. PCF sensors can be used to detect RI and temperature of solution medium. Compared with the traditional optical fiber structure, the plasma sensor based on PCF has greater advantages in coupling loss, sensor packaging difficulty and phase matching conditions [113]. PCF binds incident light using air channels or a longitudinal invariant RI structure around a hollow or solid glass core. For PCF, due to its own optical characteristics, it is easier to control the EWs generated by optical fiber. This would be a huge performance advantage for SPR and LSPR sensors [114]. According to the investigation, the SPR optical fiber sensor based on PCF is a very competitive and potential detection technology. Because of the limitation of equipment and technology, most research work based on PCF fiber is based on simulation analysis. This makes the implementation of PCF sensor structure challenging.

**Figure 7:**
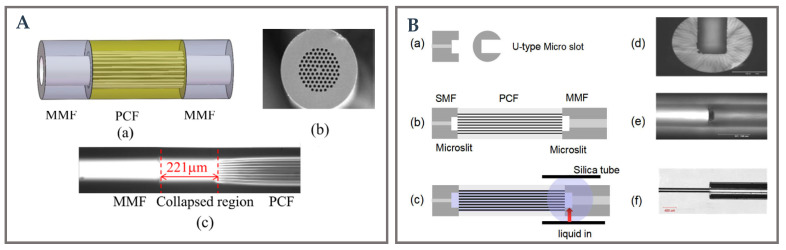
(**A**). (**a**) Schematic diagram of MMF-PCF-MMF structure, (**b**) the end face microscope diagram of PCF, (**c**) the fusing splice diagram of MMF-PCF. Reprinted with permission from Optics & Laser Technology, Copyright 2018, Elsevier [115]; (**B**). Schematic diagram of U-shaped microchips probe based on SMF-PCF-MMF fusion structure. (**a**) Side7 and top view of the fiber probe, (**b**) microslit at the splicing interface, (**c**) application of liquid detection. The microscopic images, (**d**) top view,(**e**) side view, (**f**) side view. Reprinted with permission from Optics Express, Copyright 2021, Optica [116].

Hetero-core structure is usually realized by splicing two different fibers. The mismatching of core diameter leads to the energy conversion between core and cladding modes. The strong evanescent field is produced in the cladding mode, and the evanescent field excites SPR effect when the cladding is covered by the precious metal layer. Wang et al. [115] designed a PCF-based SPR immunosensor for detecting human IgG. PCFs fused between two segments of MMF were modified with Au film, and then covered with GO and SPA for further immuno-detection. The schematic of the MMF-PCF-MMF structure is shown in Figure 7A. The results showed that the sensitivity of the sensor was 4649.8 nm/RIU, and the detection limit of human IgG was 10 ng/mL.

In fact, Yi et al. [116] designed a PCF microfluidic sensor based on four-wave mixing. Two femtosecond laser-fabricated U-shaped microchips are embedded in the sensor for real-time microfluidic measurement. This sensor is based on SMF-PCF-MMF fusion structure, and the structure diagram is shown in Figure 7B. Theoretical and experimental results reveal that the signal wavelength is sensitive to both the RI and the material dispersion properties of liquid samples placed in an air channel. The response of signal wave-length is consistent for varied low concentration water target samples. Additionally, because of the separation and dispersion characteristics of the filled liquid sample, the signal wavelength has different response with the increase of water concentration, which provides a good design method for the future wavelength-coded sensor array to recognize the liquid sample.

In recent years, the SPR fiber optic sensor based on PCF has become a research hotspot. Many researchers have realized the D-shaped [117,118,119] fiber-optic sensor based on PCF by numerical calculation and software simulation. More attention needs to be paid to developing SPR/LSPR sensors based on PCF that are more portable, cost-effective and sensitive in the future.

### 3.5. Special Fusion Splicing Structure

In hetero structure, different optical fibers are connected with each other, and there are mode field mismatch loss and conversion loss. In order to achieve effective pattern matching, transformations must be performed. In recent years, various optical fiber structures have been reported for the development of fiber-based plasma biosensors, such as tapered SMF-MMF-SMF (TSMS) fiber probes [120]. Due to its small size and all-fiber fabrication, the device is ideal for measuring gas pressure in harsh environments. Fernandes et al. [121] proposed a curvature and vibration optical fiber sensor based on the SMF-MMF-SMF-MMF-SMF (SMSMS) structure. The sensor has the characteristics of simple fabrication, low cost, high efficiency and sensitivity.

Meng et al. [122] designed a fiber-optic sensor based on MZI and SPR for RI detection by using twin-core fiber, and studied the sensing characteristics by experiments. The sensor is made by using the method of dislocation splicing. The influence of the dislocation splicing point on the MZI is analyzed systematically in this study, and a new offset splicing control method is proposed. The sensitivity is up to 3020 nm/RIU. The experimental results show that the TCF sensor has good stability. The sensor has great potential in the field of medical diagnosis, and the research has enlightening significance for the design of optical fiber devices.

### 3.6. Special Tapering Strcuture

**Figure 8:**
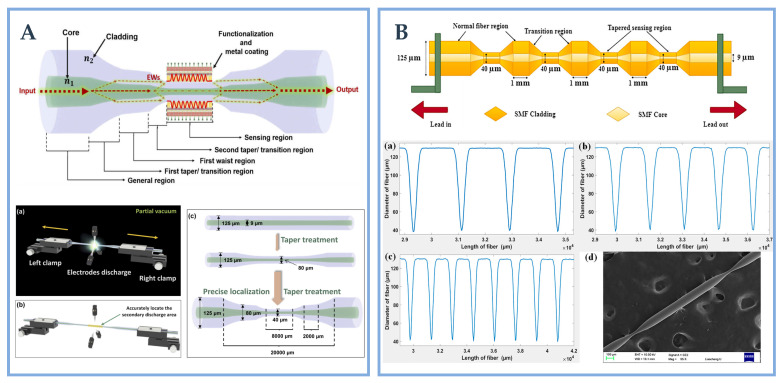
(**A**). Fabrication process of taper-in-taper structure, (**a**,**b**) fabrication process, (**c**) production process. Reprinted with permission from Optics Express, Copyright 2021, Optica [51]; (**B**). Schematic diagram of the multi-tapered fiber structure, inset is the diameter scanning results of multi-tapered fiber structure four tapers (**a**), five tapers (**b**) and eight tapers (**c**), and (**d**) SEM image. Reprinted with permission from Optics & Laser Technology, Copyright 2020, Elsevier [125].

Tapered fiber-optic has become a research frontier because of its high sensitivity, small size and easy fabrication. The traditional fabrication methods of TOF include chemical etching, oxyhydrogen flame heating and arc discharge. It is difficult to accurately grasp the position of the etching taper angle in the chemical etching method. The TOF obtained is not only poor in repeatability, but also dangerous in chemical etchants. The oxyhydrogen flame heating taper method uses oxyhydrogen flame to locally heat the optical fiber, but the loss will increase due to the introduction of impurity ions and is vulnerable to external environment interference. The arc discharge method [123] can achieve the effect of melting taper by producing high temperature through electrode discharge, which is free from external interference and has high accuracy and repeatability. The commonly used instruments for arc discharge are fiber fusion machine (FSM) and fiber combiner manufacturing system (CMS). For optical fiber SPR/LSPR sensor with tapered structure, the incident light propagates in the fiber-optic with low loss in the TIR mode. When the fiber cladding and core diameter decrease simultaneously, the TIR mode is destroyed, and the incident light diffuses part of the incident light to the cladding through the taper waist area for transmission. At this time, the light mode in the fiber-optic changes, and the basic mode propagating in the SMF will be coupled to a higher-order mode. The EWs generated by the incident light is easier to penetrate, which is conducive to the interaction between EWs and the surrounding medium, so as to achieve the purpose of detecting the changes in the surrounding RI [124]. Wang et al. [51] designed a new type with a diameter of 40 μm, which is used to detect alanine aminotransferase (ALT). On the basis of tapered optical fiber, he added taper treatment to increase the length of sensing area, thus providing more attachment sites for enzymes and nanomaterials, which is conducive to improving the sensing performance of the sensor. The taper-in-taper structure is fabricated by combiner manufacturing system (CMS) machine, and its sensing schematic, internal processing and fabrication process are shown in Figure 8A. CMS has a highly stable processing device, and its unique three electrode arc supported plasma field can provide a uniform and controllable heating range. The core of taper drawing technology is the debugging of program parameters, including the setting of electrode power, taper waist length, platform moving speed, vacuum value, etc. Through specific program control, CMS can achieve rapid and repeated drawing at precise position. Zhu et al. [125] designed a 40 μm diameter multi-taper (four-taper, five-taper, eight-taper) fiber structure for ascorbic acid detection. The multi-taper optical fiber structure is shown in Figure 8B. The fiber structure with multi-taper is developed by FSM. The melting effect is achieved by high temperature generated by double electrode discharge. The diameter of softened fiber becomes thinner under the heating of the electrode and the actuation of the motor, thus forming taper. The periodic tapered fiber can be realized at 1 mm of the normal fiber area by specific program control and setting the taper interval distance to 1 mm. The diameter distribution and SEM image of the probes are shown in Figure 8(Ba–Bd).

### 3.7. Other Special Novel Structure

**Figure 9:**
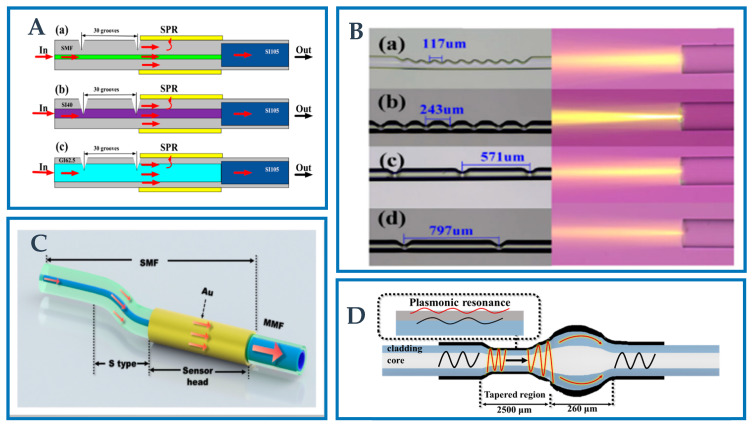
(**A**). Sensing schematic of V-groove-SMF structure (**a**–**c**). Reprinted with permission from Optics Communications, Copyright 2023, Elsevier [126]; (**B**). Schematic diagram of multi-V-groove structure (**a**–**d**). Reprinted with permission from Optics Communications, Copyright 2023, Elsevier [126]; (**C**). Schematic diagram and microscopic morphology S-shaped based SMF-MMF structure. Refer to [127]; (**D**). Schematic diagram of downward taper structure. Refer to [128].

In this part, many other novel optical fiber structures used as the SPR/LSPR sensing probe will be introduced in detail. These impressive and elaborate fiber structures provide a large number of potential strategies for the development of novel optical fiber sensors based on SPR/LSPR. Li et al. [126] proposed a novel SPR sensor based on V-groove structure, and explored the sensing results of three different optical fiber types (SMF, graded index multimode and step index multimode). The proposed structure is easy to manufacture, high sensitivity, good repeatability and high mechanical strength. The fabrication steps of the probe are shown in Figure 9A. Firstly, the bare fiber is placed on the three-dimensional workbench under the CO_2_ laser. One end of the optical fiber is fixed with an optical fiber clamp, and the other end is suspended with a light material, so that the optical fiber always maintains a constant axial stress during heating. Secondly, by adjusting the working platform, the focus of the bare fiber and the laser beam coincide. By designing the number and period of V-grooves, the CO_2_ laser is controlled by a computer to make V-grooves. The depth and period of the groove are controlled by the laser program, and the shape of the V-groove can be observed in real time through the inverted microscope. The optical fiber is deformed to form a V-groove structure under thermal action. Finally, cut off the V-groove and weld it with SMF. The micrographs of V-groove structures of different types of optical fibers are shown in Figure 9B. This novel sensor provides an important strategy for the development of SPR optical fiber sensors.

Wei et al. [127] proposed an S-shaped optical fiber strain sensor based on SPR and the structure as shown in Figure 9C. The S-shaped structure was prepared on the basis of SMF by electrofusion, and an Au film with a thickness of 50 nm was coated on the cladding surface behind the structure. The evanescent field of cladding mode contacts with the Au film to generate SPR, and the strain can change the vertical axis offset of the S-shaped optical fiber, thereby changing the incident angle of SPR, thus realizing a S-shaped optical fiber strain sensor based on SPR. Strain detection range is −1200 μϵ, and sensitivity is −14.38 pm/μϵ. The sensor provides a new method for strain measurement of fiber-optic SPR sensor.

Jin et al. [128] investigated a new LSPR sensor for RI measuring based on MMF downward taper. The schematic of the downward taper is shown Figure 9D, which is formed by splicing two SMFs into an upper taper during heating by arc discharging and pushing by the two side clamps, and the optical fibers adjacent to the top taper area are then heated and pulled to generate a lower taper. This structure effectively improves the taper ratio, thereby improving the sensitivity. First, place the two MMFs on the fiber fusion splicer platform. During the discharge, the two MMFs push each other to form a taper. Then, heating and pulling make the fiber adjacent to the upper taper area gradually thinner. The structure has small volume, low cost, simple fabrication and high mechanical strength (waist diameter 40 μm). It is anticipated that it will be employed in the field of biochemistry.

### 3.8. Other Noval Fiber Structurals

**Figure 10:**
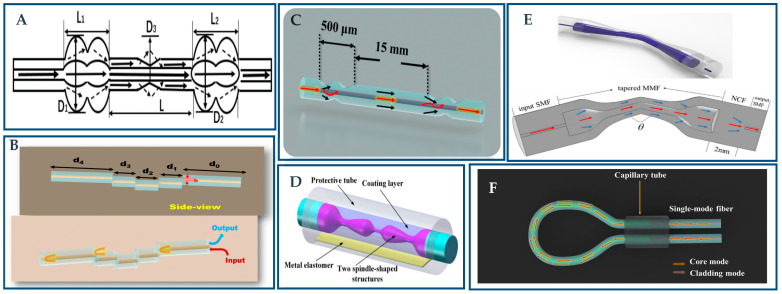
Schematic diagram: (**A**) cascaded peanut and taper structure. Reprinted with permission from *IEEE Sensors*, Copyright 2021, IEEE [129]; (**B**) schematic diagram of the off-set U-shaped optical fiber structure. Refer to [131]; (**C**) unique micro-bent core structure. Refer to [40]; (**D**) micro-fiber MZI (MMZI) with two spindle-shaped structure. Reprinted with permission from *IEEE Photonics*, Copyright 2021, IEEE [134]; (**E**) folded tapered multimode coreless fiber structure. Reprinted with permission from *Optics & Laser Technology*, Copyright 2020, Elsevier [38]; (**F**) spherical bending fiber structure. Refer to [135].

At present, many researchers have developed a variety of new optical fiber structures, but these unique structures have not been applied to optical fiber sensors based on SPR/LSPR. These structures undoubtedly provide inspiring idea for optical fiber sensing based on SPR/LSPR. Therefore, the novel optical fiber structure can be cascaded to produce a more excellent plasmonic optical fiber sensor. Lin et al. [129] designed a novel cascaded peanut and taper structure sensor based on erbium-doped fiber for temperature detection. The structure is manufactured by controlling the discharge time and current of the welding machine, as shown in Figure 10A.The peanut shape is formed by discharging at both ends of the fiber-optic to form a sphere and then splicing the two ends. The taper is formed gradually by moving the electrode after the fiber is softened due to heating during discharge. This structure has lots of advantages, including high sensitivity, ease of fabrication, high mechanical strength, compact structure, etc. Zhao et al. [130] have designed a sensor based on erbium-doped fiber with double rosette and tapered structure for RI detection. It is formed by using the welding machine to manually discharge repeatedly, making welding balls at both ends of the optical fiber, and then fusing the two balls. The taper is achieved by discharging the welding machine and moving the working platform. The double peanut like structure plays the role of beam splitting/combining. The tapered area is used as the sensing area. With the change of RI, the interference spectrum will shift. The measured RI can be obtained by monitoring the displacement. This new structure not only has good sensitivity, but also has the advantages of compact structure, easy to fabricate, low cost, etc. Fan et al. [131] proposed a new U-shaped core biased fiber-optic sensor with four optical fiber segments for strain detection. The structure and manufacturing process of the U-shaped optical fiber made of four SMF segments welded on the core offset are shown in the Figure 10B. The fabrication method is to weld four optical fiber segments with the same length together to form a U-shaped microstructure fiber. A high-precision workbench system was used in the experiment, and the fiber-optical was fixed on the high-precision platform to ensure the consistency of the probes. This unique core biased fiber has the advantages of high strain sensitivity and large strain range.

Tan et al. [132] designed a double sphere tapered fiber structure for RI, temperature and strain detection. The equipment for preparing the structure mainly uses optical fiber fusion splicer and CO_2_ laser. The preparation process is in the arc mode. When the welding joint discharges, the motor on one side moves in the opposite direction, and the softened optical fiber is extruded and expanded to a diameter of 280 μm spherical structure. Then, the same spherical structure was prepared by the same method. Finally, place the two ends of the peanut-like structure in the center of the electric platform, use the CO_2_ laser for heating, and control the electric platform to stretch the fiber 8 mm in the opposite direction at a uniform speed. Due to the advantages of simple preparation and compact structure, this structure can provide a new scheme for high-precision RI sensing fields. Ma et al. [40] designed a sensor with a unique micro bent core structure, which can simultaneously measure temperature and RI. The structure consists of two micro bending cores, as shown in Figure 10C, which are polished by high-frequency CO_2_ laser and heated by hydrogen oxygen flame. During CO_2_ laser polishing, SMF is placed under the CO_2_ laser and accurately fixed between two rotating fiber clamps. The scanning path and laser power of the CO_2_ laser are controlled by a specific program. Through different program controls and four scans, the surface of SMF becomes w-shaped. Then, the two clamps are rotated by 180° and switched to a specific program to realize the bending of the fiber core. The advantages of the designed sensor include high mechanical strength, low cost, and high sensitivity. Gang et al. [133] designed a new fiber-optic strain sensor based on SMF/HCF. The fiber-optic structure is made up of a micro-tapered SMF and micro bubble. Taper is made by melting and stretching in the discharge mode using an arc fusion splicer. Then, a section of HCF with hydrogen load is formed and spliced with SMF under arc discharge. Under discharge, the hydrogen molecules in HCF are heated and expanded to form bubbles with smooth surfaces. By repeating the above steps, the two end faces of the same SMF and HCF cascade structure are placed in the arc fusion splicer, and continuously discharge the end faces of HCF until fine bubbles are formed. Zhu et al. [134] proposed a novel micro-fiber MZI (MMZI) with two spindle-shaped structure for measuring temperature and curvature. The fiber-optic structure is shown in Figure 10D. The fabrication method is to first form a hemisphere by manually arcing one end of the fiber-optic for many times, and then weld the two optical fibers together to form the end face of the hemisphere. The size of the hemispherical end face depends on the discharge capacity and times. Subsequently, the fusion part of the optical fiber is tapered using a hydrogen oxygen flame taper machine to obtain a spindle-shaped MMZI. The thinning speed depends on the volume flow of hydrogen and oxygen. By setting parameters to precisely control the conical shape, the repeatability of MMZI preparation was ensured. The waist area is a sensing area. Increasing its length can improve the temperature sensitivity.

The structure has the benefits of low cost, high temperature, and good reproducibility, which provides new ideas for the preparation of high sensitivity sensors. Wang et al. [38] designed a novel sensor based on the folded tapered multimode coreless fiber structure for the detection of RI and temperature. The structure is shown in Figure 10E.

Its manufacturing process mainly includes two parts: fiber fusion, taper, and folding. Firstly, the SMF and MMF are coaxially connected through the fusion splicer. Then, 2 mm HCF is connected to MMF and another SMF coaxially. Taper is achieved by controlling the travel speed and heating time in the software. The structure has the advantages of simple preparation method, high sensitivity, and high mechanical strength. Tian et al. [135] proposed a new spherical bending MMF displacement sensor. The spherical bending structure is realized by bending the SMS optical fiber structure into a gas sphere with a section of capillary, and then fixing the structure shape, as shown in Figure 10F. The SMS structure is formed by splicing the 9.9 cm long MMF with two SMFs to increase the mechanical strength of the structure. Then, both ends of the SMS are inserted into the inner diameter of 1.5 cm and the length of 600 μm in capillary tubes. By moving the capillary tube, the bending radius of the spherical section can be precisely adjusted. After determining the optimal position, the optical fiber structure is fixed with UV glue. The advantages of the sensor include simple fabrication, high sensitivity, and good repeatability, etc. It is a strong candidate in the field of accurate displacement measurement. The experiment shows that the displacement sensitivity is 0.51 dB/μm.

## 4. Special Optical Fiber Structure-Based Biosensing Applications

During the past five years, the above-mentioned new structure has been widely used in actual detection, involving different biomolecules [12], chemicals [13], and temperature [14]. This detection function can be used in a wide range of applications such as the water environment pH value and RI [15], various gases [16], and humidity [17]. In this section, the application of the plasma sensor based on the special optical fiber structure is introduced for biomolecular detection, chemical quantities detection, and physical quantities detection. In recent years, many studies on the applications of special optical fiber structures have been summarized to help facilitate understanding of related research progress as depicted in Table 2, Table 3 and Table 4.

### 4.1. Biomolecular Detection

Optical fiber biosensors are favored because of their advantages such as no electromagnetic interference, acid and alkali corrosion resistance, and miniaturization structure. According to the signal conversion mode of the sensor, the fiber optic biosensors more studied are the fiber EWs sensor, SPR sensor, fluorescence quenching sensor, and fiber grating sensor [18,136]. The optical fiber biosensor structure mainly includes light source, optical fiber, bio-sensitive element, and signal detection system. The bio-sensitive element is the key component of the sensor, and the commonly used bio-sensitive element mainly includes antigen, antibody, enzyme, and nucleic acid. The tested object selectively interacts with specific bio-sensitive elements (i.e., specific binding of antigen antibody or receptor ligand; complementary pairing of nucleic acid molecular bases; specificity of enzyme action on substrate, etc.), and the physical characteristics of light transmitted in the generated biochemical information modulation optical fiber, such as light intensity, light amplitude, phase, etc. Therefore, this kind of sensor has strong selectivity and high sensitivity, but the complex formed or the spectral behavior generated by the organism is similar, which cannot be distinguished by the optical fiber alone. Indicators or markers, such as enzymes, fluorescent substances, acid and alkali indicators, are often used. Compared with other biosensors, optical fiber biosensors combine the characteristics of optical fiber sensing, which is specifically reflected in the following benefits: (1) Due to the good insulation and shielding effect of the optical fiber itself, it has strong anti-interference ability and is not disturbed by surrounding electromagnetic fields. (2) The probe can be miniaturized and operated conveniently. (3) It can realize telemetering and real-time, online and dynamic detection. (4) Fast response and high sensitivity.

**Figure 11:**
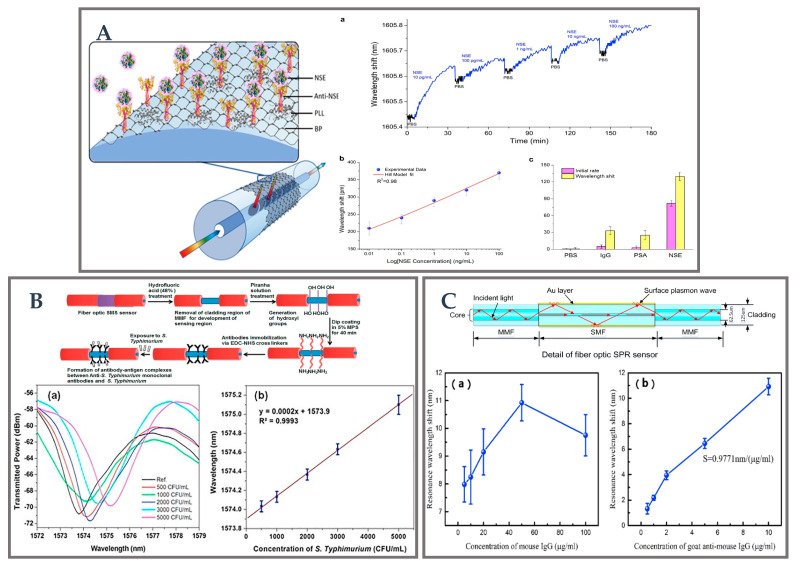
(**A**). The schematic of BP-TFG, inset (**a**) wavelength shift with different concentration of NSE samples at 0.01, 0.1, 1.0, 10, and 100 pg/mL, respectively, (**b**) linearity plot and (**c**) selectivity test. Reprinted with permission from *Biosensors and Bioelectronics*, Copyright 2019, Elsevier [137]; (**B**) the functionalize process of SMS, inset (**a**) quantity detection results of *Salmonella typhimurium*, (**b**) linearity plot. Reprinted with permission from *Optical Fiber Technology*, Copyright 2018, Elsevier [140]; (**C**). The schematic of MSM, inset (**a**) wavelength shifts under different M-IgG-decorated concentrations, (**b**) wavelength shifts about detecting different concentrations of GAM-IgG. Reprinted with permission from *Biosensors and Bioelectronics*, Copyright 2019, Elsevier [72].

The first black phosphorus (BP)-fiber-optic biosensor was proposed by Zhou et al. for the ultrasensitive detection of human neuron-specific enolase (NSE) cancer biomarkers [137], which has been shown in Figure 11A. This new optical nano-configuration, in which BP is biofunctionalized by poly-l-lysine as a key crosslinker, has been exploited to promote the bio-nano-photonic interface with extremely strong light-matter interaction. The developed biosensor is mainly comprised of anti-NSE immobilized BP nanosheets with the most tilted fiber Bragg gratings (BP-TFG). In this work, they demonstrated extremely high sensitivity with LOD as low as 1.0 pg/mL. It created a bio-nano-photon platform based on a novel fiber structure for their application in biomedical research, environmental monitoring, food safety, and other fields. Similarly, a label-free fiber-optic biosensor for the real-time detection of the inflammatory marker c-reactive protein (CRP) in serum was developed and tested by Esposito et al. [138]. The authors showed the brand-new arrangement of a long-period grating (LPG) in a double-clad fiber with a particular RI curve. Through mode switching and a mild chemical etching of the fiber surface, the sensitivity has been enhanced. A broad working range of clinically significant CRP values was explored, and serum was shown to have a low detection limit of 1.1 ng/mL. Esposito et al. then announced the development of an engraved unique double-clad fiber-based long-period grating (LPG) biosensor for vitamin D detection [139]. The mode conversion phenomena caused by the chemical etching of the fiber outer coating increases its sensitivity. In the case of hetero-core-structured fibers, the varied structural characteristics of neighboring cores will lead to differing transmission constants. This will cause a phase mismatch between cores, thus lowering the transverse coupling between cores. Further, Kaushik et al. employed a label-free optical fiber biosensor based on SMS optical fiber construction for detection of bacteria [140], as shown in Figure 11B. Its primary objective is to accurately identify *Salmonella typhimurium* in samples of spiked milk and phosphate buffered saline (PBS). For biosensor applications, a multi-mode interference-based SMS sensor was developed. Typhoid monoclonal antibody-functionalized SMS biosensors can efficiently detect *Salmonella typhimurium* in the linear range of 500 to 5000 CFU/mL. Its sensitivity was found to be 275.86 nm/RIU, with an LOD value of 247 CFU/mL, as seen in the inset (a) and (b) of Figure 11B. The suggested biosensor technology may be employed for sensitive pathogen detection, and this study’s effort provides an alternative platform for the quick and accurate detection of *Salmonella typhimurium*. Cardiac troponin I (cTnI) as a barometer of heart health is an important substance involved in the regulation of myocardial contraction. To detect the cardiac troponin I (cTnI) solution, Wang et al. also created an SMS hetero-core optical fiber sensor construction based on LSPR [141]. AuNPs and ceria nanoparticles (CeO_2_-NPs) were used to fix the fiber structure to improve its sensing ability. The reaction between cTnI solutions of different concentrations and mAb-cTnI leads to the change in RI value in the medium around the probe, which leads to the shift in peak wavelength. The proposed sensor probe in the detection range of 0–1000 ng/mL is 3 pm/(ng/mL), and the LOD is 108.15 ng/mL, respectively. Later, Wang et al. modified the improved MMF photosensitive optical fiber MMF (MPM) optical fiber structure with GO, AuNPs, and MoS_2-_NPs to improve the sensitivity and stability of the sensor probe [142]. Additionally, the sensing surface has been functionalized with enzymes to improve its selectivity. The final performance index of the sensor is that the sensitivity is 3.4 pm/(ng/mL) within the linear range of 0–1000 ng/mL, and the LOD 96.2638 ng/mL, which is slightly improved compared with the sensing performance of the SMS structure. The sensor can be widely used in the field of real-time detection of acute myocardial infarction (AMI).

Similar to MPM, Mai et al. proposed modifying the structure of MMF-SMF-MMF (MSM) with Au membrane and antigen fragments with an SPR biosensor probe [72] (as shown in Figure 11C). The sensing results are shown in inset (a) and (b) of Figure 11C. The clear shift in resonance spectrum indicates that the monitor capacity of the target antibody was enhanced due to the increase in the modified concentration of M-IgG from 0 to 50 ng/mL. When the concentration of analytes was more than 50 g/mL, the particular detection layer was thickened, and the detection effectiveness was decreased. This is caused by the random self-assembly that resulted in the accumulation of oversaturated M-IgG fragments. The antigen-modified fiber probe has a sensitivity and LOD of 0.9771 nm/(g/mL) and 0.1 g/mL, respectively. In another study, Li et al. successfully developed a new LSPR fiber biosensor for the detection of creatinine based on the hetero-cores structure of SMF-MCF-MMF-SMF [50]. Zhu et al. have also made remarkable achievements in the detection of acetylcholine by using the hetero-core structure biosensor probe constructed by MMF-MCF-MMF [104].

A highly birefringent superfine fiber Bragg grating with temperature compensation was proposed by Xiao et al. [143]. The effective recording of high reflectivity FBG is made possible by the significant energy overlap offered by MMF. Although the ambient RI sensitivity is much different from that of the double resonances obtained from the orthogonal polarization state, this difference allows for temperature-compensated RI detection. The covalent functionalization approach was used to immobilize human immunoglobulin G (IgG) molecules on the surface of the micro-fiber grating probe to specifically detect anti-IgG molecules. This technique offers a high-efficiency and low-cost design for micro-fiber Bragg grating-based biosensors without taking into account temperature cross-sensitivity. A taper interferometer implanted in FBG was proposed by Sun et al. for their application in label-free detection of breast cancer biomarkers (HER2) [144]. The resonant wavelengths of the fiber grating have not been sensitive to changes in RI, whereas the tapered fiber interferometer sensor is quite sensitive to ambient RI. Due to its independent reaction to temperature, FBG may be used as a thermometer to track undesirable drift. By conjugating the HER2 antibody using the covalent immobilization approach, the precise detection of the target biomarker was made possible in the label-free biometrics program. The developed sensor offers a platform for early breast cancer diagnosis with a low LOD of 5 ng/mL.

**Figure 12:**
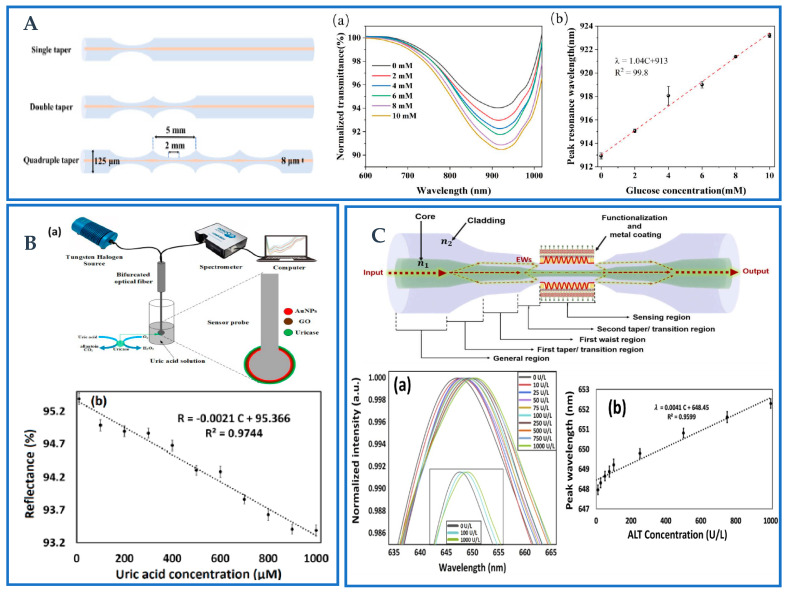
(**A**). The schematic of quadruple-taper biosensors, inset (**a**) wavelength shifts about the detection of different concentration of glucose solution and (**b**) related linearity plot. Reprinted with permission from *IEEE Sensors*, Copyright 2022, IEEE [145]; (**B**). micro-ball structure used for the uric acid solution, inset is related linearity result. Reprinted with permission from *IEEE Trans Nanobioscience*, Copyright 2020, IEEE [71]; (**C**). The schematic of taper-in-taper structure, inset (**a**) wavelength shifts about the detection of different concentrations of ALT and (**b**) linearity plot. Reprinted with permission from *Optics Express*, Copyright 2021, Optica [51].

Li et al. developed novel continuous quad-taper biosensors to achieve accurate detection of the glucose level in the human body [145], as shown in Figure 12A. The inset of Figure 12A shows examples of sensing results for glucose solution (a) normalized transmittance, and (b) linearity of the synthetic sensor. The sensitivity and LOD of the probe structure were 1.04 nm/mM and 0.24 mM, respectively, in the linear range from 0 to 10 mM. Its potential for measuring glucose in the human body has also been demonstrated. Zhu et al. developed fiber-optic biosensing probes based on the LSPR phenomenon using AuNPs and ZnO nanoparticles (ZnO-NPs) coated tapered SMF [146]. The sensitivity of the probe is 5.7 nm/mM in the linear range of 10–200 μM, and the LOD is 25.78 μM. On the basis of this study, Zhu et al. used AuNPs and GO-modified sensing probes developed multiple-tapered (four-tapered, five-tapered, and eight-tapered) optical fiber sensors for ascorbic acid detection [125]. Through the comprehensive comparison of sensitivity, LOD, and other parameters, the tapered fiber probe seems to have better performance. The result shows that the sensitivity (8.3 nm/mM), correlation coefficient (0.9724), and LOD (51.94 μM) of the sensor are better than those of the previously designed sensors.

Takhmina et al. proposed a spherical resonator based biosensor for thrombin prototype detection [84]. To quantify different protein concentrations, the gadget employs quick and accurate CO_2_ laser splicing, followed by gold sputtering and functionalization. An optical backscatter reflectometer that measures the reflectance spectrum probes the spherical resonator, which functions as a weak interferometer with an echo loss below −50 dB. Here, we provide a sample with high sensitivity (1273.74 nm/RIU), which enables the detection of proteins at a concentration between 0.4 and 100 pM, and a log-response LOD of 1.56 pM. In another study, Kumar et al. proposed a high sensitivity and selectivity fiber-optic sphere LSPR biosensor. In the proposed sensor, a microsphere fiber optic sensor probe was designed to fabricate a 350 μm diameter fiber optic sphere coated with AuNPs and GO using an advanced splicing machine to improve its sensitivity [71]. The sensor structure and test device diagram are shown in Figure 12B. The sensor probe was functionalized to detect uric acid (UA) with specific enzymes such as uricase. Uricase/GO/AuNPs coated optical fiber sensors have shown detection in the linear range of 10–1 mM. The reflectivity of the sensor decreases linearly with the increase in the UA concentration, as shown in Figure 12B inset. The sensitivity of the sensor is 2.1 %/mM and the LOD is 65.60 μM. The findings demonstrate that the sensor may be utilized to detect UA in human serum samples. It can detect a small quantity of UA solution because of the tiny sensor probe. Overall, the sensor has a wide linear range and low detection limit, is inexpensive, has great accuracy, and is sensitive and selective to a wide variety of signals. A quick bio-optical sensor was created by Park et al. that precisely diagnoses infectious diseases by using a ball-lensed optical fiber (BLOF) probe and an automated analysis platform [147]. The LSPR concept was used by Wang et al. to develop a new biosensor for the detection of ALT analytes at concentrations ranging from 0–1000 U/L [51]. According to Figure 12C, an optical fiber construction with a tapered-in-tapered shape has been developed for the first time specifically for biosensing applications. The developed sensor has been produced using a kind of arc discharging technology with three-electrode under semi-vacuum conditions. The average normalized wavelength spectra from three different fiber probes are shown in Figure 12C inset. The linearity of the graph is 0.9562, indicating that the shift of the resonance peak linearly increases with the increasing concentration of the ALT enzyme. According to the data analysis, the sensitivity of the sensor is 4.1 points/(U/L) and thus can effectively be used in the diagnosis of human liver injury.

**Table 2:** Application of special optical fiber structure in biomolecular detection.

Characteristics	Nanomaterials	Measured Parameter	Sensing Range /LOD	Sensitivity	Ref.
SMF–MCF–MMF–SMF	GO/AuNPs /MoS_2_-NPs/CA	Creatinine	0–2000 μM /128.4 μM	0.0025 nm/μM	[50]
Micro-ball	Au	Protein	0.4–100 pM	1273.74 nm/RIU	[84]
Convex fiber-tapered seven core fiber-Convex fiber (CTC)	AuNPs /Nb_2_CTx Mxene /CA	Creatinine	86.12 μM	3.1 pm/μM	[103]
Tapered	AuNRs/AuTNPs/ AuNPs	microRNA	103 aM–261 aM	0.92 nm/nM 0.97 nm/nM	[148]
U-shaped	Au/miRNA-133a	mRNA	0.0133 ng/mL	27.352 dB/log ng/mL	[149]
Tapered POF	molecularly imprinted polymer	L-nicotine	n.r. ^a^	1.3 × 10^4^ nm/M −1.7 × 10^3^ nm/M	[150]
U-bent fiber	Au layer/ITO nanorods /graphene	DNA	0.1–100 nM/0.10 nM	690.7 nm/RIU	[151]
D-shape plastic optical fiber	Graphene/Au	DNA	n.r. ^a^	1227 nm/RIU	[152]
D-shaped POF	HMM/ graphene	DNA	10 pM–100 Nm /10 pM	0.26 nm/ nM	[153]
SMF-NCF_SMF	Au	cDNA	80 nm	n.r. ^a^	[154]
Tapered SMS	AgNPs/GO	L-Cysteine	10 nM–1 mM/63.25 μM	7.0 nm/ mM	[155]
TFBG	Au/ graphene	Dopamine	10^−13^–10^−8^ M /10^−13^ M	0.29 dB/ logM	[156]
Tapered	AgNPs	Dopamine	0.58 μM	n.r. ^a^	[157]
U-shaped MMF-MSM	Au	IgG	0.104 μg/mL	0.192 nm/ wt./mL	[158]
Dual channel	Au layer/GO /anti-IgG/ AuNPs-IgG	IgG	1–35 μg/mL /0.015 μg/mL	1.36 nm/(μg/mL)	[159]
MMF-NCF-MMF structure	Polyelectrolyte self-assembled multilayers	IgG	1.75 μg/mL	57.06 nm /(mg/mL)	[160]
Tapered	AuNPs	Uric acid	280.07 μM	0.0131 nm/μM	[161]
SMF and TFBG	Au/AuNPs	Thrombin	1 nm	n.r. ^a^	[162]
SMF and TFBG	Au	Formaldehyde	0–17 ppm	2.10 pm/ppm	[163]

^a^ not reported.

### 4.2. Chemical Quantities Detection

Chemical applications in almost all types of plasma biosensing applications, such as the preparation of the probe, requires nano-material fixation and preparation of the reagent to be tested. A chemical reaction occurs at the probe surface during the detection of various biomolecules. Therefore, the chemical and biological applications are inseparable. The application of plasma sensors in chemical and biochemical processing is to monitor the complex changes in the chemical reaction process in real time through the RI measurement of analytes, which is of great significance in environmental monitoring, food monitoring, gas detection, heavy metal detection, pharmaceutical medicine, public health, biotechnology, and other fields. The detection system is used to turn chemical information from chemical analysis into useful signals for real-time monitoring. Satyendra et al. [13] proposed a novel optical fiber probe based on the no-cladding-core structure, which was immobilized with a layer of indium tin oxide (ITO) and bromocresol purple (BCP) for ammonia detection. The results showed the sensitivity of 1.891 nm/ppm in the range of 1–10 ppm [13]. Bijoy et al. [164] utilized a U-shaped fiber structure based on a large-core POF to measure the concentration of Pb^2 +^ in water. The U-shaped optical fiber has high sensitivity and point illumination, and its bending structure can reduce the difficulty of fixed materials. To increase the sensitivity and specificity of the sensor, they mounted oxalic acid-functionalized AuNPs on the surface of the probe sensing area. A photodetector measures the output voltage, power, and intensity of the sensor system to determine the change in lead ion concentration. According to the findings, the sensor has an excellent linear fit in the linear range of 1–20 ppb and LOD of 2.1 ppb, which is substantially lower than the recommendation of 10 ppb. Further, a highly sensitive SPR-pH sensor with a core diameter of 600 μm was proposed by Sarika et al. and based on a high RI layer and intelligent hydrogel [165]. In order to improve the coupling of light in the optical fiber, the SPR optical fiber probe was sharpened at both ends. The resonance wavelength of an aqueous solution shows blue shift as its pH value rises, and as the pH of the liquid rises, so will the measurement accuracy. Li et al. created a layer-by-layer self-assembled fiber probe for pH detection based on the unique fusion structure of MMF-HCF-MMF. [166]. The sensor has good stability, the detection range is from 3.18 to 11.84, and the average sensitivity is 32.31 nm/pH.

**Table 3:** Application of special optical fiber structure in chemical quantities detection.

Characteristics	Nanomaterials	Measured Parameter	Sensing Range/LOD	Sensitivity	Ref.
Unclad fiber	ITO/BCP	Ammonia gas	1~10 ppm	1.891 nm/ppm	[13]
U-shaped	Au	Pb^2+^	2.1 ppb	n.r. ^a^	[164]
D-shaped	Au	Pb^2+^	n.r. ^a^	0.116 nm/ppm	[167]
MMF-SMF-MMF	Hydrogel/Au	pH	8–10 pH	13 nm/pH	[168]
SMF-FBG	Cu/WS_2_/PAAG	pH	1–9	−4.42 nm/pH	[169]
TFBG	Ag	H_2_O_2_	0.2 μM	n.r. ^a^	[170]
Unclad fiber	Cu/ZnO	H_2_S gas	n.r. ^a^	n.r. ^a^	[171]
SMF-MMF-SMF	Onic liquid gel coatings	CO_2_ gas	n.r. ^a^	n.r. ^a^	[172]
MMF-Tapered HCF-MMF	Au	RI	1.335–1.40	7592.25 nm/RIU	[173]
Tapered and U-shaped (MMF-TUMMF-MMF)	Au	RI	n.r. ^a^	X: 1415/RIU y: 1293/RIU	[174]
D-shaped	Graphene/Ag	RI	n.r. ^a^	9000 nm/RIU 14,500 nm/RIU	[175]
D-shaped	Au	RI	n.r. ^a^	30 nm/RIU	[176]
U-shaped	Au/PML	RI	1.33–1.44	66,000 nm/RIU	[177]
D-shaped	Au/self-assembled monolayer	Cu^2+^	4 × 10^−6^–2 × 10^−4^ M	n.r. ^a^	[178]
MMF-NCF-MMF	Au	Cu^2+^	n.r. ^a^	0.1184 nm/μM	[179]

^a^ not reported.

### 4.3. Physical Quantities Detection

Materials with improved sensing performance and detection ability in challenging situations have been used in the development of fiber optical sensors. However, the typical photoelectric mechanism struggles to match the detection requirements in several unique situations that call for high sensitivity and resolution. Thus, new sensor kinds must be investigated in order to resolve the numerous issues with monitoring structures in severe environmental conditions. This section mainly introduces the application of some physical parameters (such as humidity, temperature, magnetic field, curvature, etc.) of optical fiber sensors.

**Figure 13:**
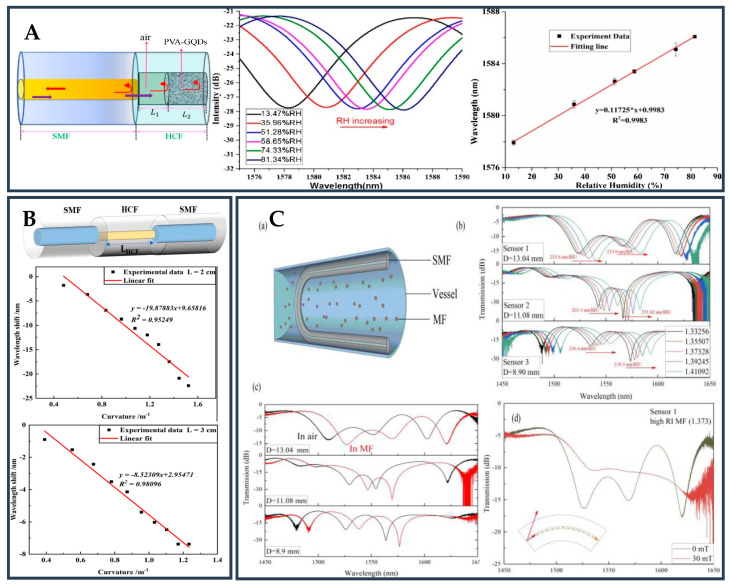
Schematic diagram and related results of the novel fiber. (**A**). Fabry-Perot interferometer (FPI) used for the monitoring of RH, and on the right part shows the related results (wavelength shifts due to the changes in RH values and related linearity plot). Reprinted with permission from *Sensors and Actuators* B: Chemical, Copyright 2019, Elsevier [108]; (**B**). SMF-HCF-SMF structure used for identifying the changes in curvature and the related linearity results with HCF lengths of 2 cm and 3 cm, respectively. Reprinted with permission from *Optical Fiber Technology*, Copyright 2021, Elsevier [180]; (**C**). (**a**) Schematic diagram of vector magnetic field sensor based on U-shaped structure. (**b**) Transmission spectra about the quantity detection within different RIs of solutions. (**c**) Transmission spectra about the detection with different samples: in air (black line) and in magnetic field (red line). (**d**) Transmission spectra in magnetic field with high RI (1.373) under 0 and 30 mT magnetic field. Reprinted with permission from *Optics Express*, Copyright 2021, Optica [181].

Humidity sensors, as the name suggests, measure the concentration of water vapor in the air, which has been widely used in the monitoring of environmental humidity, food protection, environmental detection, greenhouse farming, and other aspects of important applications. Zhao et al. [108] proposed and experimentally demonstrated a new fiber Fabry-Perot interferometer (FPI) based on graphene quantum dots (GQDs) and polyvinyl alcohol (PVA) for relative humidity (RH) monitoring. As seen in Figure 13A, an HCF that is spliced at the end of an SMF is filled with the GQDs-PVA compound. The length of the FP cavity elongates and the RI of the GQDs-PVA compound lowers with an increase in relative humidity. This change causes the reflectance spectra to shift toward the length wavelength and may be used to identify changes in RH values. Different saturated brine solutions provide the humidity environment, and a hygrometer is used to calibrate the RH value. According to the experimental findings, as indicated in the inset of Figure 13A, the relative humidity varies from 0.1374–0.8134 RH.

The resonant spectrum and relative humidity value’s fitting line exhibits good linearity. The findings indicate that the linear correlation is 0.9983 and humidity sensitivity is 117.25 pm/% RH. It has a promising future for practical development.

Yang et al. proposed a high-sensitivity optical fiber curvature sensor [180] based on SMF and HCF, using a SMF-HCF-SMF multimode interference (MMI) structure as shown in Figure 13B. By connecting different SMFs together, a tiny inner diameter HCF can be quickly and simply constructed to form a sensor. Multiple orientation modes may be directly stimulated on the sidewalls of the HCF without the need for any extra structure since the inner diameter of the HCF is less than the core diameter of the SMF. Then, curvature may be determined by keeping an eye on the transmission spectrum’s shift as a result of the bend-sensitive MMI effect. The experimental results show that a high sensitivity similar to 19.88 nm/m^−1^ can be achieved in the range of 0.48~1.52 m^−1^ curvature.

Magnetic field sensors are now an essential fundamental part of information technology and the information industry in the modern information society. Magnetic field sensors are currently extensively employed in scientific study, manufacturing, and social life in all facets of applications, as shown by examining various types of data. A new type of vector magnetic field sensor with compact structure and easy fabrication is proposed and studied by Li et al., as shown in Figure 13(Ca). The proposed sensor consists of a U-shaped SMF fixed in a container filled with magnetic fluid [181]. There is no need for extra fiber Bragg gratings or mechanical modification during the sensor production process. The results show that by using a mode supported by a U-shaped bending fiber structure with an appropriate bending radius, it is possible to assess the direction and strength of the magnetic field using the reaction of magnetic fluid to the magnetic field. The maximal magnetic field intensity sensitivity was 0.517 nm/mT, while the directional sensitivity was 0.251 nm/°. The results of the testing demonstrate the configuration’s strong production reproducibility. Experimental measurements of the U-bend SMF’s RI response was made and the results are discussed as follow. The transmission spectrum exhibits a number of interference obliquities. According to Figure 13(Cb), sensor 2 showed the deepest interference inclination at around 1550 nm and the best bending diameter, indicating that all three sensors were most sensitive to light in the cladding mode. All interference is red-shifted as the RI of the external solution increases. The sensitivity of all dip is around 200–240 nm/RIU in the range of 1500–1600 nm. The RI of magnetic field in this investigation was around 1.357. Figure 13(Cc) displays the equivalent transmission spectra of the U-bend SMF sensor in air and MF. Higher concentrations of magnetic field (bigger RI) may be more magnetic field sensitive. However, due to cladding mode leakage in U-bend fibers in magnetic field with higher RI, a considerable interference decrease may not be seen at a very high magnetic field. As seen in Figure 13(Cd).

**Figure 14:**
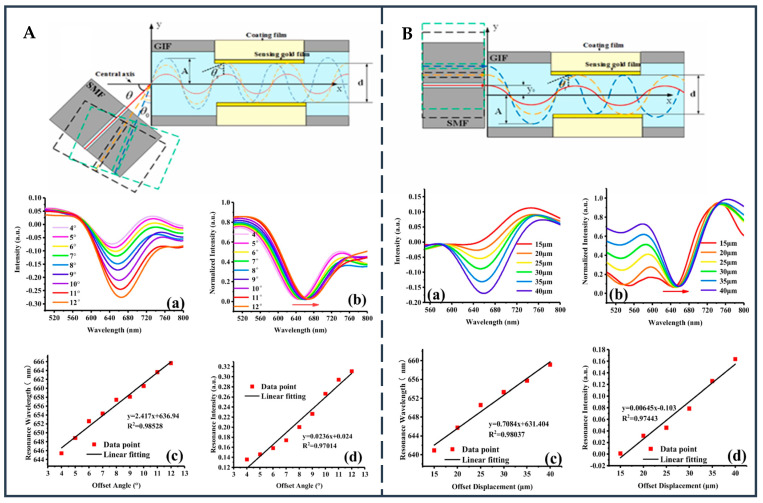
(**A**). The schematic diagram of micro-angle measurement sensor probes, inset (**a**,**b**) incident light deviation angle detection results, and (**c**,**d**) linearity plot. Reprinted with permission from *Sensors and Actuators* A: Physical, Copyright 2022, Elsevier [184]; (**B**). The schematic diagram micro-displacement measurements sensor probes, inset (**a**,**b**) deviation displacement detection results, and (**c**,**d**) linearity plot. Reprinted with permission from *Sensors and Actuators A*: Physical, Copyright 2022, Elsevier [184].

The generation and development of temperature sensors is essential as temperature is a physical quantity used to indicate the degree of cooling and heating of an object; it is one of the basic testing parameters in industrial automation, household appliances, environmental protection, safety production and the automobile industry. Chiang et al. explored theoretically and experimentally how temperature can affect the dielectric constant of the metal film, thus changing the position of the resonant valley. However, results show very low sensitivity [182]. A novel all-optical fiber temperature sensor was created by Mohanraj et al. employing MoS_2_ nanoplates coated in D-shaped fibers (DSF). Strong simultaneous field interaction and superb optical absorption in DSF and MoS_2_ nanoplates produce extremely high sensitivity. The maximum output power of 7 dB is attained when the suggested all-fiber temperature sensor is investigated in the temperature range of 26–83 °C. The results of the experimental investigation demonstrate the high repeatability of the MoS_2_ based all-fiber sensor. The sensitivity of the suggested temperature sensor may alternatively be computed as 0.1211 dB/°C based on the two fitting curves. As a result, the suggested optical fiber sensor may be employed in difficult environments to monitor dynamic temperature [183]. An essential tool for assessing the safety of geotechnical constructions including slopes, dams, tunnels, and excavations is the prediction of displacement or strain. Because of its evident benefits, such as lightweight, high precision, strong durability, wide measuring range, and long transmission distance, optical fiber displacement sensor has currently received a lot of attention. Liu et al. developed a dual-parameter SPR sensor for micro-angle and micro-displacement measurements using gradient index MMF [184]. Intensity of light traveling through the sensor varies with the micro-angle and micro-displacement, as shown in Figure 14A,B, respectively. Light is injected into a graded-index multimode fiber using a conventional SMF.

The gradient index MMF has a unique RI distribution that causes the beam path to be sinusoidal. Figure 14A shows that when the deviation angle grows, the transmission beam’s phase changes, the beam path’s amplitude rises, and the TIR angle (SPR incidence angle) lowers. Evanescent field is produced at the contact between the fiber core and gold film. The resonance valley of SPR may be seen in the output spectrum when the propagation frequency of EWs and SPWs are the same. As seen in Figure 14A, the angular displacement table is adjusted to raise the deviation angle from 4–12°, which controls the axial deviation of the SMF along the central axis. The depth of the sensor’s resonance valley shifts to the long wavelength with an increase in incident light deviation angle, as shown in Figure 14(Aa,Ab). The probe’s sensitivity to light intensity and wavelength is 0.236 A and 2.417 nm/° as shown in Figure 14(Ac,Ad), respectively. Similarly, authors changed the two displacement tables to regulate the vertical movement of the SMF along the *y*-axis, with the deviation displacement rising from 15 to 40 μM, in order to restore the SMF to its initial position using the angular displacement table. The resonant wavelength and intensity of the resonant light grows when the deviation displacement increases, as illustrated in Figure 14(Ba,Bb). The probe has a wavelength sensitivity of 0.708 nm/° and a light intensity sensitivity of 0.006 A, respectively as shown in Figure 14(Bc,Bd).

**Table 4:** Application of special optical fiber structure in physical quantities detection.

Characteristics	Nanomaterials	Measured Parameter	Sensing Range/LOD	Sensitivity	Ref.
Tapered MMF bent into loop	Au	Load	0–20 kPa	1.47 nm/kPa	[34]
SMF-GI MMF-SI MMF-SMF-SI MMF	Au	RI/Temperature	0–25 nm 20–60 °C	DSR: 4.24 nm/μm −0.19 nm/°C TSR: 0.46 nm/μm −2.485 nm/°C	[37]
MMF-PCF-MMF	Au/PDMS	Temperature	35–100 °C	−1.551 nm/°C	[40]
Double-side polished U-shape POF	Au/PDMS	RI/Temperature	n.r. ^a^	1258 nm/RIU –0.596 nm/°C	[185]
MMF-SMF-MMF	Au	RI/Temperature	n.r. ^a^	2323.4 nm/RIU 2.850 nm/°C	[186]
SMF-NCF-SMF	Au	RI/Temperature	2061.6 nm	2061.6 nm/RIU −0.0379 nm/°C	[187]
Two opposite D–shaped	Au/Ethanol/polyvinyl alcohol	Temperature/Humidity	10–70 °C, 20–80% RH	2.9 nm/°C 11.6 nm/%RH	[188]
Single mode-side polished multimode-singlemode (SSPMS)	gelatin material	Humidity	40–90 %RH	0.14 dB/%RH	[189]
Spiral twisted LPFG	Au/WS_2_	Humidity	n.r. ^a^	37.3 pm/% RH	[190]
MMF-SMF	Au	Temperature/Salinity	n.r. ^a^	−4.418nm/°C 0.558 nm/‰	[191]
MMF-SMF	Au	Temperature/Salinity	n.r. ^a^	−2.02 nm/°C 0.31 nm/‰	[192]
MMF-PCF-SMF	Au	Temperature/Pressure	n.r. ^a^	−1.802 nm/°C 2.838 nm/MPa	[193]
C-type micro-structured fiber	Au	Temperature/Salinity	5–35 °C 30–40‰	−7.609 nm/°C 1.402 nm/‰	[194]

^a^ not reported.

## 5. Future Prospects

Practical real-time application is the ultimate goal of any scientific research. Optical fiber sensors based on plasma have been widely used in biology, medicine, food, and environmental monitoring. Through the use of special probe structure, the LSPR-based optical fiber sensor can be applied to the direct detection of molecules with very small diameters.The LSPR effect of novel metal nanoparticles can be well applied to the direct detection of molecules with very small diameter. The SPR sensor has a wide range of applications, such as biomolecular detection, hazard detection, catalytic chemical kinetics, physical parameter monitoring and other fields, and plays a huge role in real-time monitoring of chemical kinetics. Still now, the promising detection technology has obviously not been maximized in the application of optical fiber sensing, which is greatly limited by the optical fiber treatment process. For example, SPR sensor based on PCF is a competitive, potential, and advanced detection technology. However, due to the limitation of manufacturing level, most of the research work is still in the stage of numerical simulation especially PCF-based sensor’s work. As one aspect of the future, more attention needs to be paid for development of PCF-SPR sensors that are simpler, more cost-effective, more reliable and easier to manufacture. Multiple parameters simultaneous detection scheme of optical fiber biosensor based on plasma has always been an important direction for researchers to explore. Additionally, the special optical fiber processing technology is still a work in progress; many novel optical fiber structures will be designed to inject new vitality in the future. Of course, the key to these design ideas is how to make the core mode as close as possible to the special metal mode and external media mode, so as to more effectively stimulate the SPP. The future development direction based on the existing structure may pay more attention to the processing of fiber core and fiber plane, which puts forward a higher throughput micro–nano manufacturing technology and material fixation technology. Some of the more advanced micro-and nano-fabrication technologies, such as femtosecond laser processing technology [26], will continue to improve the processing of fiber microstructure and greatly reduce the difficulty of manufacturing these sensing structures.

Meanwhile, in the face of complex environments and diverse conditions, sensor characteristics such as stability, sensitivity, specificity and reproducibility have always been important criteria for evaluating the performance of sensors. These necessary indicators are the premise of actual detection, and also an important direction of sensor development now and in the future. To solve this problem, the combination of plasma resonance technology and other detection technologies like directional resonance coupling (DRC), wavelength division multiplexing (WDM) [195], time division multiplexing (TDM) [196], long-range surface plasmon resonance (LRSPR) [197], quantum plasmon [198] etc. to form a multi-technology integrated detection system may be a promising direction of development. In addition to these processes, researchers should look into possible solutions for the packaging of sensor probes and commercialization of their products on the market.

Additionally, simultaneous measurement of multiple parameters, application of nanomaterials, miniaturization and portability of packaging schemes are also the future development directions. A forward-looking idea is to give special treatment to the structure of the optical fiber probe, so as to design multiple sensing areas covered with different excitation materials for the detection of different analytes. Here, it is required that the functionalization materials have obviously different photosensitive properties. For example, different thicknesses of gold and silver films have different resonance wavelengths, but this also puts forward strict requirements for the material immobilization process. Another great challenge is that the demodulation of the sensing signal is extremely difficult. Tabassam et al. put forward a sensor to measure two parameters at the same time in 2016 [199]. In that work, they cascaded the two SPR sensing probes, using the different excitations of the metal film to produce a special valley of SPR resonance effect. Fortunately, this detection method is being validated in clinical analysis, and also provides ideas for simultaneous detection of multiple parameters in the future. At present, it is another challenge for biosensors to detect multiple parameters in one optical fiber device. This requires consistent design and optimization of the probe structure. Although the difficulty of material coating is expected to be greatly reduced, response time and reusability are still important challenges.

## 6. Conclusions

This paper summarizes the promising plasma sensors based on the combination of SPR/LSPR technology and optical fiber technology. The fabrication process, principle and design idea of the optical fiber structure are mainly summarized, and its feasibility is fully verified by examples. Because each sensor structure has its advantages, the appropriate sensor structure can be selected according to specific needs, so that researchers can use it as a reference for research and development. From the perspective of optical fiber structure, the research results of optical fiber biosensors based on SPR/LSPR in recent years are summarized in detail. This paper has collected the recently impressing plasmonic optical fiber structures, and reported their parameters, principles and applications one by one. Then, a large number of studies on the application of biosensors based on special fiber structures are summarized from three perspectives, and the future development is prospected. At present, the field is striving to move from laboratory research to commercial application, and the pipeline is as long as possible. Starting from interdisciplinary academic fields and innovative development ideas, the vigorous development of plasma optical fiber biosensors needs the attention and participation of more field personnel. It is hoped to arouse readers’ interest in plasma sensing technology and jointly deal with the challenges brought by the demand for high-quality sensing.

## Figures and Tables

**Table 1:** Summary of classical special fiber structures recently proposed.

	Characteristics	Fabrication Method	Materials	Sensitivity	Ref.
FBG	TFBG	Ultraviolet laser phase mask technique	Au	−6721 dB/RIU	[75]
	TFBG	Scanning phase mask technique and laser	Au	510.5 nm/RIU	[76]
	TFBG	n.r. ^a^	Au	100 nm/RIU	[77]
	TFBG	Laser irradiation through a phase mask	Au	n.r. ^a^	[78]
Tapered optical fiber	Tapered MMF	Flame brush method	Platinum	7.04 μm/RIU	[79]
	Double-layer uniform-waist tapered fibers	Heat fiber to melting point then stretch in opposite directions	Al/TiO_2_	2400 nm/RIU	[80]
	Tapered SMF	Heat and stretch with a motorized linear positioning stage	AuNPs/GO	1.06 nm/mM	[81]
	Tapered SMF	Fiber-optic taper element technology	Ag	n.r. ^a^	[82]
	Tapered SMF	n.r. ^a^	Au	2021.07 nm/RIU	[83]
Micro-ball	Micro-ball	CO_2_ laser splicer	Au	1273.74 nm/RIU	[84]
Hetero-core fiber	SMF-EPCF-SMF	n.r. ^a^	-	4.16 nm/°C	[85]
	SMF-HCF-SMF	n.r. ^a^	Au	1037 nm/RIU	[86]
	SMF-HCF-SMF	n.r. ^a^	Au	2061.6 nm/RIU	[87]
	SMF-thin core fiber-SMF	n.r. ^a^	-	18.3 pm/°C	[88]
D-shaped optical fiber	D-shaped	Femtosecond laser system	Polydimethylsiloxane	521.92 nm/RIU	[89]
	D-shaped	Side polishing technique	MoS_2_-graphene composite	6708.87 nm/RIU	[90]
	D-shaped	Side polishing technique	ITO	60,000 nm/RIU	[91]
	D-shaped	Wheel polishing technology	Two nanoscale gold belts	1700–12600 nm/RIU	[92]
	D-shaped	Wheel polishing and side polishing technology	TiO_2_ layer with Au nanoparticles embedded	0.83 nm/(g/L)	[93]
U-shaped optical fiber	U-shaped	Burn into U-shape	Au and Bi_2_O_2_Se	6827.41 nm/RIU	[94]
	U-shaped	Fiber fixed to rubber column	TiO_2_/Au	5959 nm/RIU (n_d_ = 1.333) 11,092 nm/RIU (n_d_ = 1.383)	[95]
	U-shaped	Etch using hydrofluoric acid	Polyimide–silica hybrid film	n.r. ^a^	[96]
	Double U-shaped	n.r. ^a^	Au	0.172 nm/μg/mL	[97]

^a^ not reported.

## Data Availability

Not applicable.
